# Decreased Expression of Cell Adhesion Molecule 4 in Gastric Adenocarcinoma and Its Prognostic Implications

**DOI:** 10.3390/diagnostics12040941

**Published:** 2022-04-09

**Authors:** Seongsik Bang, Seungyun Jee, Hwangkyu Son, Hyebin Cha, Jongmin Sim, Yeseul Kim, Hosub Park, Jaekyung Myung, Su-Jin Shin, Hyunsung Kim, Seungsam Paik

**Affiliations:** 1 Department of Pathology, Seoul Hospital, Hanyang University College of Medicine, Seoul 04763, Korea; grypony@naver.com (S.B.); jee.seung.yun@gmail.com (S.J.); ganzi4900@gmail.com (H.S.); chbin0111@gmail.com (H.C.); parkhstm@gmail.com (H.P.); tontos016@naver.com (J.M.); 2 Department of Pathology, Anam Hospital, Korea University College of Medicine, Seoul 02841, Korea; j07star2@gmail.com (J.S.); coabee@hanmail.net (Y.K.); 3 Department of Pathology, Gangnam Severance Hospital, Yonsei University College of Medicine, Seoul 06273, Korea; charm@yuhs.ac

**Keywords:** gastric adenocarcinoma, cell adhesion molecule 4, immunohistochemistry, prognosis

## Abstract

Cell adhesion molecule 4 (CADM4) is a novel tumor suppressor candidate. The prognostic implications of CADM4 in gastric cancer have not been conclusively elucidated. Therefore, we evaluated the clinicopathological significance and prognostic value of CADM4 expression in a large series of patients with gastric adenocarcinoma. Immunohistochemical staining for CADM4 was performed on 534 gastric adenocarcinomas. We evaluated the associations between CADM4 expression and the clinicopathological and molecular characteristics of the adenocarcinomas. The prognostic effect of CADM4 expression was evaluated by survival analyses. Low CADM4 expression was significantly associated with young age (*p* = 0.046), aggressive histological type (*p* < 0.001), high pT category (*p* < 0.001), nodal metastasis (*p* < 0.001), high stage (*p* = 0.002), lymphovascular invasion (*p* = 0.001), and perineural invasion (*p* = 0.001). Low CADM4 expression was more frequently observed in tumors without human epidermal growth factor receptor 2 (HER2) amplification (*p* = 0.002). Low CADM4 expression was associated with worse overall survival (*p* = 0.007) and recurrence-free survival (*p* = 0.005) in the survival analyses. Low CADM4 expression was associated with aggressive clinicopathological features and poor clinical outcomes. CADM4 can act as a tumor suppressor in gastric adenocarcinoma and can be considered a prognostic biomarker.

## 1. Introduction

Gastric cancer is a leading cause of cancer-related deaths in the world. In 2020, gastric cancer was the fifth most common type of newly diagnosed cancer cases and the fourth most common cause of cancer-related death [1]. Although the incidence and mortality rates of gastric cancer are gradually decreasing worldwide, there are many new cases in East Asia including Korea and Japan [2]. Gastric cancer is a heterogeneous group that can be divided based on distribution and histological patterns. In addition, gastric cancer can be classified according to molecular drivers, and the molecular classification published by The Cancer Genome Atlas (TCGA) research network is representative [3]. Each molecular subtype is associated with clinical characteristics such as the prognosis following surgery, benefit from chemotherapy, and the effect of anti-programmed cell death-1 (PD-1) antibodies [4]. Clinical research on a novel therapy targeting various biomarkers is underway, such as anti-PD-1 antibodies (for tumors with microsatellite instability high phenotype or expressing programmed cell death-ligand 1) and trastuzumab (for tumors with HER2 positivity) used in routine clinical practice [4].

Cell adhesion molecule 4 (CADM4/TSLL2/SynCAM4/Necl-4/IGSF4C) is one of the immunoglobulin (IG) superfamily molecules and has structural similarity to tumor suppressor in lung cancer 1 (TSLC1) [5]. TSLC1 is a novel tumor suppressor gene for non-small cell lung cancer [6]. CADM4 shows significant homology with TSLC1, including a short cytoplasmic domain that is considered to play a critical role in tumor suppressor activity [7]. Therefore, there have been several studies on CADM4 expression in cancer cells and its role in the development or progression of cancer. In vitro studies on several types of cancer, including prostate cancer, glioma, colon cancer, renal cell carcinoma, and lung cancer, showed that CADM4 was overall down-regulated [7,8,9,10,11]. In addition, overexpression of CADM4 was efficient in suppressing the tumorigenicity of cancer cells [8,9,11]. Similar to these results, a decrease in or loss of expression of CADM4 in tumor cells was associated with aggressive clinicopathological phenotypes and poor prognosis, suggesting the potential of CADM4 as a tumor suppressor [10,12,13,14,15,16,17,18].

For gastric cancer, Song et al. reported that a lower expression level of CADM4 mRNA was associated with aggressive features [19]. However, Sayar et al. could not reveal a significant relationship between CADM4 expression and clinicopathological characteristics in gastric cancer [16]. Therefore, in this study, we investigated the associations between CADM4 expression and clinicopathological features of gastric cancer by performing immunohistochemical (IHC) staining on a large series of resected samples. In addition, the associations between CADM4 expression and molecular characteristics including HER2 status were evaluated.

## 2. Materials and Methods

### 2.1. Patient Selection and Clinicopathological Data Collection

A total of 544 patients with gastric adenocarcinoma were enrolled retrospectively in this study. All patients underwent surgical resection (gastrectomy or submucosal dissection) between February 2005 and August 2010 at Hanyang University Hospital in the Republic of Korea. We excluded 10 cases due to incomplete follow-up data or no available tumor tissue and conducted the study with the remaining 534 cases. All tissue slides used at the time of diagnosis and pathologic reports were reviewed by two pathologists (Bang, S. and Shin, S.-J.). We assessed pathologic features of tumor location, gross type, histological type, Lauren classification, lymphatic or vascular invasion, perineural invasion, pT stage, and pN stage. The histological type was assessed using the World Health Organization classification for gastric adenocarcinoma, and the pathologic stage was assessed using the 8th edition of the American Joint Committee on Cancer (AJCC). Medical records were reviewed to obtain clinical information, including patient age, sex, and follow-up data.

### 2.2. Tissue Microarray Construction and Immunohistochemical Staining

Tissue microarray (TMA) construction was performed using a Tissue Microarray Set (Labro, Seoul, Korea) and formalin-fixed paraffin-embedded tissues. All gastric cancer tissues used in the study were resection specimens, and a representative portion of the tumor was selected by light microscopy. Then, we obtained a 3.0 mm tissue core from the donor block and transferred it to the recipient block (Labro, Seoul, Korea). Each TMA consisted of 6 × 5 samples.

We cut 4-μm-thick sections from each TMA block. IHC staining for CADM4 was performed with a Benchmark XT automated staining system (Ventana Medical Systems, Tucson, AZ, USA). Primary antibody against CADM4 (1:100, SAB4500746, Sigma-Aldrich, St. Louis, MO, USA) was used according to the manufacturer’s instructions.

### 2.3. Interpretation of Immunohistochemical Staining

IHC staining assessment was performed by two pathologists (Bang, S. and Paik, S.) without access to the clinical data. For semi-quantitative assessment, cytoplasmic staining of the tumor cells was assessed using the immunoreactive score (IRS) as previously described [12]. The intensity of staining was categorized as 0 to 3 (0: negative, 1: weak, 2: moderate, and 3: strong), and the proportion of staining was graded as 0 (0%), 1 (1–25%), 2 (26–50%), 3 (51–75%), and 4 (>75%). IRS was calculated as the product of the intensity and proportion of staining and ranged from 0 to 12. A receiver operating characteristics (ROC) curve analysis was used to determine optimal cutoff value. We divided the cases into low expression (IRS ≤ 4) and high expression (IRS > 4) groups, and these were used for all statistical analyses.

### 2.4. Assessment of Molecular Characteristics

The TCGA Research Network proposed dividing the molecular classification of gastric cancer into four molecular subtypes: Epstein–Barr virus (EBV), microsatellite instability (MSI), genomic stability (GS), and chromosomal instability (CIN) [20]. To identify the MSI subtype, we performed IHC staining on representative tumor sections. Four primary antibodies against mismatch repair proteins were used: MLH1 (G168-728, Cell Marque, Rocklin, CA, USA), PMS2 (MRQ-28, Cell Marque, CA, USA), MSH2 (G219-1129, Cell Marque, CA, USA), and MSH6 (PU29, Leica Biosystems, Nussloch, Germany). If one or more mismatch repair proteins was not expressed in all tumor cells, it was considered a negative result and classified as an MSI subtype. EBV-encoded RNA in situ hybridization (EBER-ISH) using an INFORM EBER Probe (Roche, Basel, Switzerland) was performed, and cases showing diffuse positivity in tumor cells were classified as EBV subtype. GS and CIN subtypes were not distinguished in our study.

IHC staining and silver DNA in situ hybridization (SISH) were used to determine HER2 status. Automatic staining was performed with anti-HER2 antibody on the whole tumor sections according to the manufacturer’s instructions (4B5, Roche, Basel, Switzerland) using the INFORM HER2 Dual ISH DNA probe cocktail (Roche, Basel, Switzerland). We considered HER2-amplified cases as those with strong membranous reactivity (3+) in IHC staining or with a HER2–chromosome 17 ratio ≥ 2.0 in SISH [21].

### 2.5. Statistical Analyses

All data were analyzed using SPSS software version 25.0 (IBM, Armonk, NY, USA). Pearson’s chi-square test was performed to evaluate the correlations between CADM4 expression and clinicopathological characteristics and between CADM4 expression and molecular characteristics. The Kaplan–Meier method with the log-rank test was used to identify the influence of CADM4 expression on overall survival (OS) and recurrence-free survival (RFS). The Cox proportional hazard model was used to determine the significant prognostic factors. A two-tailed *p*-value < 0.05 was considered statistically significant.

## 3. Results

### 3.1. The Baseline Characteristics of Gastric Adenocarcinoma Patients

The median age of the patients was 60 years (range: 25–90), and the male/female ratio was 2.32:1. Among the 534 patients, 45 (8.4%) underwent submucosal dissection, 253 (47.4%) received gastrectomy alone, and 236 (44.2%) received gastrectomy with adjuvant chemotherapy. In total, 103 (19.3%) patients received fluoropyrimidine-based chemotherapy, 6 (1.1%) patients received platinum-based chemotherapy, and the remaining 127 (23.8%) patients received combination therapy with fluoropyrimidine and platinum. No patients received targeted therapy (including trastuzumab). None of the patients who underwent submucosal dissection had clinically suspected lymph node metastasis. Of the patients, 340 (63.7%) were diagnosed with early gastric cancer (pT1–2) and the remaining 194 patients (36.3%) were diagnosed with advanced gastric cancer (pT3–4). No lymph node metastasis was observed in 318 patients (59.6%, Nx + pN0). The clinicopathological characteristics of the selected cases are summarized in Table 1.

### 3.2. Correlations between CADM4 Expression and Clinicopathological Characteristics

**Figure 1:**
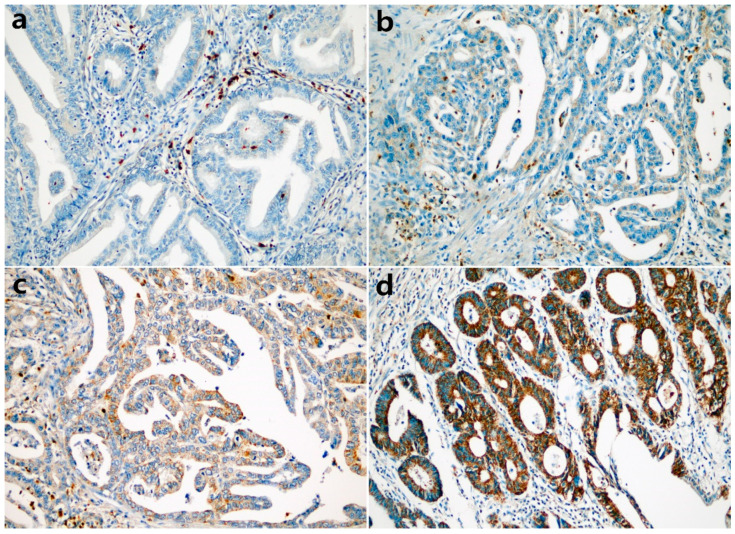
Representative photomicrographs of immunohistochemical staining with CADM4 in gastric adenocarcinoma ((**a**): negative, (**b**): weak, (**c**): moderate, (**d**): strong, ×200).

The cytoplasmic expression of CADM4 was detected, and representative photomicrographs are presented in Figure 1. CADM4 expression was reduced or absent in 303 of 534 gastric adenocarcinomas. Low CADM4 expression was significantly associated with young age (*p* = 0.046), undifferentiated and other histologic types (*p* < 0.001), diffuse and mixed types of Lauren subtype (*p* < 0.001), high pT category (*p* < 0.001), nodal metastasis (*p* < 0.001), high stage (*p* = 0.002), lymphovascular invasion (*p* = 0.001), and perineural invasion (*p* = 0.001). The patient’s sex and tumor location were not significantly associated with CADM4 expression. The correlations between CADM4 expression and clinicopathological characteristics are summarized in Table 2.

### 3.3. Correlations between CADM4 Expression and Molecular Characteristics

There was no significant correlation between CADM4 expression and the 35 (6.6%) cases of EBV subtype or the 43 cases (8.1%) of MSI subtype. We identified 26 HER2 amplification cases (4.9%) that were not included in the EBV and MSI subtypes. Low CADM4 expression was more frequently observed in the tumors without HER2 amplification and was statistically significant (*p* = 0.002, Table 3).

### 3.4. CADM4 Expression and Prognostic Implication

**Figure 2:**
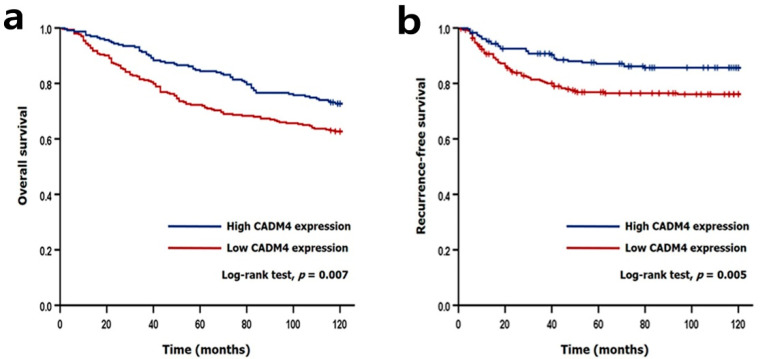
Kaplan–Meier analyses for overall survival (**a**) and recurrence-free survival (**b**). Overall survival and recurrence-free survival were significantly worse in patients with low CADM4 expression (Log-rank test, *p* = 0.007 and *p* = 0.005, respectively).

**Figure 3:**
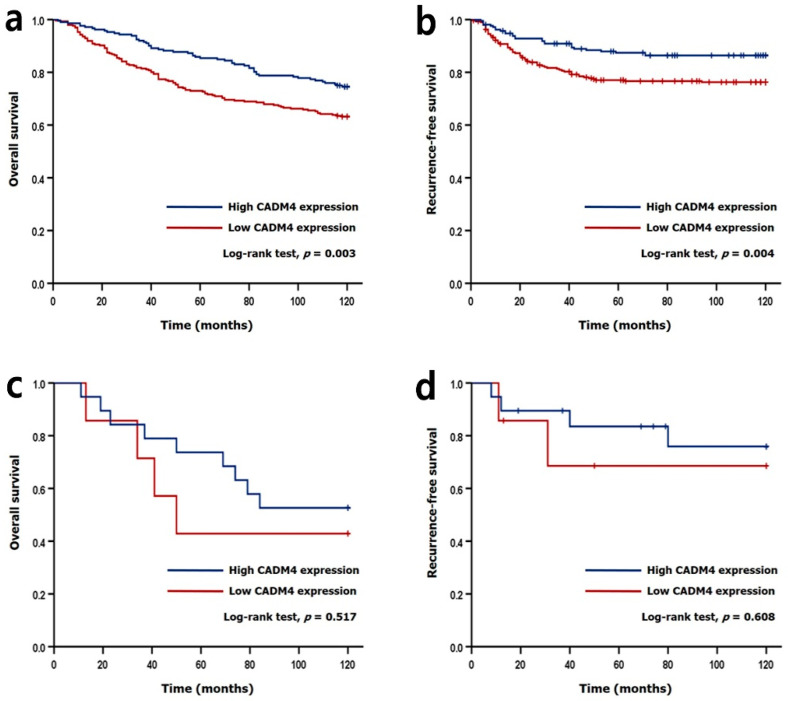
Kaplan–Meier analyses in the HER2-negative group (**a**,**b**) and the HER2-positive group (**c**,**d**). Overall survival and recurrence-free survival were significantly worse in patients with low CADM4 expression in the HER2-negative group (Log-rank test, *p* = 0.003 and *p* = 0.004, respectively). In the HER2-positive group, there was no significant difference in overall survival or recurrence-free survival according to CADM4 expression.

We performed Cox regression analyses to investigate the significant clinicopathological factors and to reveal the prognostic significance of CADM4 expression (Table 4). In univariate analyses, low CADM4 expression (*p* = 0.007), old age (*p* < 0.001), diffuse and mixed types of Lauren classification (*p* = 0.019), high stage (*p* < 0.001), lymphovascular invasion (*p* < 0.001), perineural invasion (*p* < 0.001), and HER2 amplification (*p* = 0.049) were associated with short OS. Low CADM4 expression (*p* = 0.006), undifferentiated and other histological types (*p* < 0.001), diffuse and mixed type of Lauren classification (*p* < 0.001), high stage (*p* < 0.001), lymphovascular invasion (*p* < 0.001), and perineural invasion (*p* < 0.001) were associated with short RFS. There was no prognostic difference according to MSI subtype and EBV subtype (data not shown). In multivariate analyses, low CADM4 expression (*p* = 0.002), old age (*p* < 0.001), high stage (*p* < 0.001), perineural invasion (*p* = 0.030), and HER2 amplification (*p* = 0.010) were associated with short OS, and high stage (*p* < 0.001) was associated with short RFS. The Kaplan–Meier method was used to reveal the prognostic significance of CADM4 expression. Patients with low CADM4 expression showed significantly shorter OS and RFS (log-rank test, *p* = 0.007 and *p* = 0.005, respectively; Figure 2) compared to those with high CADM4 expression. In the HER2 non-amplified cases, patients with low CADM4 expression had shorter OS and RFS (log-rank test, *p* = 0.003 and *p* = 0.004, respectively). However, CADM4 expression did not significantly affect OS and RFS in patients with HER2 amplification (Figure 3).

## 4. Discussion

In this study, we performed IHC staining with CADM4 antibody on 534 gastric adenocarcinomas. We evaluated the associations of CADM4 expression with clinicopathological features, prognostic significance, and molecular characteristics. Low expression of CADM4 was significantly associated with aggressive histological type, high pT category, nodal metastasis, high stage, lymphovascular invasion, and perineural invasion. The results suggest that CADM4 down-regulation is associated with aggressive clinicopathological features. Low CADM4 expression is an independent prognostic factor for OS by univariate and multivariate Cox regression analyses. Low CADM4 expression was associated with worse RFS in univariate analysis. However, it lost statistical significance in multivariate analysis. Using Kaplan–Meier survival analyses, we determined that low CADM4 expression was associated with poor prognosis. Low CADM4 expression was not associated with EBV or MSI subtype.

CADM4 is a potential prognostic biomarker and is thought to play a role as a tumor suppressor. Jang et al. showed that the loss of CADM4 expression is relatively frequent in colorectal adenocarcinomas and proposed that CADM4 plays an important role in cancer progression and patient survival [12]. Jang et al. demonstrated that a loss of or decrease in CADM4 expression likely plays an important role in breast cancer invasiveness and is associated with worse biological parameters [13]. Kawanishi et al. suggested that negative Necl-4 expression is associated with carcinogenesis and the aggressiveness of pancreatic ductal adenocarcinomas [14]. Kim et al. reported that the loss of CADM4 expression is associated with poor prognosis in patients with small intestinal adenocarcinoma [15]. Luo et al. showed that down-regulation of CADM4 promotes tumor growth and metastasis in non-small cell lung cancer [11], and Du et al. predicted CADM4 as a therapeutic target [22].

The mechanism regulating CADM4 expression in tumors is unknown. According to the TCGA’s Pan-Cancer Atlas studies, genetic alterations of CADM4 have been reported in only a small number of cases (2.38%). Several studies have revealed that TSLC1, which has structural similarity to CADM4, is down-regulated by promoter methylation in some tumors (30–60%) [23]. CADM4 expression is also irreversibly down-regulated in cancer cells in several studies, and there is a possibility of a decrease in CADM4 expression due to DNA methylation [24,25], but further study is needed.

Two previous studies have reported the expression of CADM4 in gastric cancer. Song et al. demonstrated that the expression of CADM4 is down-regulated in gastric cancer tissues and cell lines, and that down-regulation of CADM4 in patients without lymph node metastasis is significantly associated with the degree of cell differentiation, depth of tumor invasion, lymph node metastasis, and TNM stage [19]. Sayar et al. performed IHC staining on 51 gastric cancer tissues and reported that expression of CADM4 at the protein level does not show a significant association with tumor differentiation, lymphovascular invasion, perineural invasion, depth of tumor invasion, vascular invasion, or metastasis [16]. In our study, we evaluate CADM4 expression in a large series of patients with gastric adenocarcinoma. Our study reveals that low CADM4 expression is significantly associated with aggressive clinicopathological features and poor prognosis. The relationships between CADM4 expression and molecular characteristics also are evaluated, and low CADM4 expression is more frequently observed in HER2-negative tumors.

In conclusion, we demonstrate the associations between low CADM4 expression and aggressive clinicopathological features and clinical outcomes for gastric adenocarcinoma. CADM4 can act as a tumor suppressor in gastric adenocarcinoma. Future studies are needed to define the molecular mechanisms regulating the expression of CADM4 and its usefulness as a therapeutic target.

## Figures and Tables

**Table 1:** Baseline characteristics of selected cases (*n* = 534).

Clinicopathologic Characteristics	Case No. (%)
Age, median (range, year)	60 (25–90)
Sex	
Male	373 (69.9%)
Female	161 (30.1%)
Tumor size, mean (range, cm)	4.1 (0.3–20.0)
Location (center of tumor)	
Cardia	15 (2.8%)
Fundus	2 (0.4%)
Body	177 (33.1%)
Angle	23 (4.3%)
Antrum	309 (57.9%)
Pylorus	8 (1.5%)
Gross type (early gastric cancer)	
Type I	15 (5.1%)
Type IIa	28 (9.6%)
Type IIb	36 (12.3%)
Type IIc	159 (54.5%)
Type III	15 (5.1%)
Mixed	39 (13.4%)
Borrmann type (advanced gastric cancer)	
Borrmann type 1	5 (2.1%)
Borrmann type 2	52 (21.5%)
Borrmann type 3	156 (64.5%)
Borrmann type 4	29 (12.0%)
Histologic type	
Tubular adenocarcinoma, well differentiated	82 (15.4%)
Tubular adenocarcinoma, moderately differentiated	129 (24.2%)
Tubular adenocarcinoma, poorly differentiated	114 (21.3%)
Papillary adenocarcinoma	2 (0.4%)
Mucinous adenocarcinoma	10 (1.9%)
Poorly cohesive carcinoma (including signet ring cell carcinoma)	107 (20.0%)
Other histologic subtypes *	21 (3.9%)
Mixed adenocarcinoma	69 (12.9%)
Lauren classification	
Intestinal	228 (42.7%)
Diffuse	135 (25.3%)
Mixed	153 (28.7%)
Indeterminate	18 (3.4%)
Lymphovascular invasion	
Present	264 (49.4%)
Not identified	270 (50.6%)
Perineural invasion	
Present	199 (37.3%)
Not identified	335 (62.7%)
T category	
pT1a	177 (33.1%)
pT1b	115 (21.5%)
pT2	48 (9.0%)
pT3	101 (18.9%)
pT4a	86 (16.1%)
pT4b	7 (1.3%)
N category	
Nx	45 (8.4%)
pN0	273 (51.1%)
pN1	57 (10.7%)
pN2	62 (11.6%)
pN3a	46 (8.6%)
pN3b	51 (9.6%)
Stage (AJCC 8th edition)	
IA	260 (48.7%)
IB	43 (8.1%)
IIA	53 (9.9%)
IIB	33 (6.2%)
IIIA	50 (9.4%)
IIIB	44 (8.2%)
IIIC	51 (9.6%)
Treatment	
Submucosal dissection	45 (8.4%)
Gastrectomy	253 (47.4%)
Gastrectomy + fluoropyrimidines Gastrectomy + platinum compounds Gastrectomy + fluoropyrimidines + platinum compounds	103 (19.3%) 6 (1.1%) 127 (23.8%)

* Other histologic subtypes, carcinoma with lymphoid stroma, hepatoid adenocarcinoma, micropapillary adenocarcinoma.

**Table 2:** Correlations between CADM4 expression and clinicopathological characteristics in patients with gastric adenocarcinoma (*n* = 534).

Variables	CADM4 Expression		*p*-Value
	Low Expression (%) (*n* = 303)	High Expression (%) (*n* = 231)	
Age			0.046
<65 years	195 (60.2%)	129 (39.8%)	
≥65 years	108 (51.4%)	102 (48.6%)	
Sex			0.283
Female	97 (60.2%)	64 (39.8%)	
Male	206 (55.2%)	167 (44.8%)	
Location			0.845
Proximal	109 (56.2%)	85 (43.8%)	
Distal	194 (57.1%)	146 (42.9%)	
Histologic type *			< 0.001
Differentiated	77 (36.2%)	136 (63.8%)	
Undifferentiated and others	226 (70.4%)	95 (29.6%)	
Lauren classification			< 0.001
Intestinal	81 (35.5%)	147 (64.5%)	
Diffuse and mixed	210 (72.9%)	78 (27.1%)	
Indeterminate	12 (66.7%)	6 (33.3%)	
pT category			< 0.001
pT1 and pT2	173 (50.9%)	167 (49.1%)	
pT3 and pT4	130 (67.0%)	64 (33.0%)	
Nodal status			< 0.001
Negative	157 (49.4%)	161 (50.6%)	
Positive	146 (67.6%)	70 (32.4%)	
Stage			0.002
I	154 (50.8%)	149 (49.2%)	
II and III	149 (64.5%)	82 (35.5%)	
Lymphovascular invasion			0.001
Not identified	135 (50.0%)	135 (50.0%)	
Present	168 (63.6%)	96 (36.4%)	
Perineural invasion			0.001
Not identified	172 (51.3%)	163 (48.7%)	
Present	131 (65.8%)	68 (34.2%)	

* Differentiated: well differentiated tubular adenocarcinoma, moderately differentiated tubular adenocarcinoma, papillary adenocarcinoma; undifferentiated: poorly differentiated tubular adenocarcinoma, poorly cohesive carcinoma; others: mucinous adenocarcinoma, other histologic subtypes; stage, AJCC 8th edition.

**Table 3:** Correlations between CADM4 expression and molecular characteristics in patients with gastric adenocarcinoma (*n* = 534).

Variables	CADM4 Expression		*p*-Value
	Low Expression (%) (*n* =303)	High Expression (%) (*n* = 231)	
EBV status			
Negative	283 (56.7%)	216 (43.3%)	0.960
Positive	20 (57.1%)	15 (42.9%)	
MSI status			
MSS	282 (57.4%)	209 (42.6%)	0.275
MSI	21 (48.8%)	22 (51.2%)	
HER2 status			
No amplification	296 (58.3%)	212 (41.7%)	0.002
Amplification	7 (26.9%)	19 (73.1%)	

Abbreviations: EBV, Epstein–Barr virus; MSS, microsatellite stable; MSI, microsatellite instability; HER2, human epidermal growth factor receptor-2.

**Table 4:** Univariate and multivariate Cox regression analyses among gastric adenocarcinoma patients (*n* = 534).

Overall Survival						
Variables	Univariate Analysis			Multivariate Analysis		
	HR	95% CI	*p*-Value	HR	95% CI	*p*-Value
CADM4 expression (high vs. low)	1.523	1.119–2.073	0.007	1.695	1.215–2.367	0.002
Age group (<65 vs. ≥65)	1.932	1.437–2.597	<0.001	2.064	1.520–2.801	<0.001
Histologic type *	1.301	0.956–1.771	0.094			
Lauren classification ^†^	1.446	1.062–1.970	0.019	1.208	0.850–1.572	0.292
Stage (I vs. II, III)	3.996	2.902–5.502	<0.001	2.659	1.572–4.498	<0.001
LVI (not identified vs. present)	3.088	2.236–4.265	<0.001	1.197	0.736–1.948	0.468
PNI (not identified vs. present)	3.486	2.575–4.719	<0.001	1.659	1.051–2.620	0.030
HER2 amplification (negative vs. positive)	1.763	1.002–3.102	0.049	2.135	1.197–3.809	0.010
**Recurrence-Free Survival**						
**Variables**	**Univariate Analysis**			**Multivariate Analysis**		
	**HR**	**95% CI**	***p-*** **Value**	**HR**	**95% CI**	***p-*** **Value**
CADM4 expression (high vs. low)	1.802	1.186–2.737	0.006	1.299	0.842–2.005	0.237
Age group (<65 vs. ≥65)	0.973	0.651–1.453	0.892			
Histologic type *	2.966	1.819–4.837	<0.001			
Lauren classification ^†^	3.558	2.181–5.806	<0.001	1.387	0.822–2.342	0.221
Stage (I vs. II, III)	15.873	8.258–30.510	<0.001	6.708	2.644–17.019	< 0.001
LVI (not identified vs. present)	11.721	6.099–22.524	<0.001	2.236	0.917–5.454	0.077
PNI (not identified vs. present)	8.349	5.162–13.505	<0.001	1.543	0.847–2.810	0.156
HER2 amplification (negative vs. positive)	1.274	0.588–2.906	0.566			

* Histologic type: differentiated vs. undifferentiated and others; ^†^ Lauren classification, intestinal vs. diffuse and mixed; stage, AJCC 8th edition. Abbreviations: HR, hazard ratio; 95% CI, 95% confidence interval; LVI, lymphovascular invasion; PNI, perineural invasion; HER2, human epidermal growth factor receptor 2.
